# Tax-induced rapid senescence is mediated by both classical and alternative NF-κB pathways

**DOI:** 10.1186/1742-4690-8-S1-A189

**Published:** 2011-06-06

**Authors:** Yik-Khuan Ho, Huijun Zhi, Dominic DeBiaso, Hsiu-Ming Shih, Chou-Zen Giam

**Affiliations:** 1 Department of Microbiology and Immunology, Uniformed Services University of the Health Sciences, Bethesda, MD, 20814, USA; 2 Institute of Biomedical Sciences, Academia Sinica, Taipei, 115, Taiwan

## 

HTLV-1 oncoprotein, Tax, is a potent activator of classical and alternative NF-κB signaling pathways and is thought to promote cell proliferation and transformation via NF-κB activation. We have shown recently that hyper-activation of NF-κB by Tax immediately triggers the cellular senescence checkpoint. Down-regulation of NF-κB activation, by contrast, rescues cells from Tax-induced rapid senescence (Tax-IRS). Here, we demonstrate that the IKKalpha and NEMO subunit of the IKK complex are absolutely essential to Tax-IRS. Repression of IKKalpha or NEMO expression by small hairpin RNAs abrogated Tax-mediated activation of both classical and alternative NF-κB pathways and rendered the knockdown cells resistant to Tax-IRS. IKKalpha deficiency moderately affected Tax-induced I-κB-alpha degradation and NF-κB nuclear localization, but drastically repressed NF-κB transcriptional activation. While IKKbeta and TAK1 knockdown significantly attenuated Tax-induced NF-κB transcriptional activation respectively, they only partially prevented Tax-IRS and did not display significant effect on the alternative pathway. Importantly, NIK knockdown yielded similar effects as IKKalpha and NEMO knockdown. These data suggest that Tax, through its interaction with NEMO, help recruit NIK and TAK1 for IKKalpha and IKKbeta activation respectively. Tax-mediated senescence as driven by the NEMO/NIK/IKKalpha complex involves both classical and alternative NF-κB pathways. Thus, the quality rather than the quantity of NF-κB activity is more important for promoting senescence.

